# A Crosslinked Soybean Protein Isolate Gel Polymer Electrolyte Based on Neutral Aqueous Electrolyte for a High-Energy-Density Supercapacitor

**DOI:** 10.3390/polym11050863

**Published:** 2019-05-13

**Authors:** Pengfei Huo, Shoupeng Ni, Pu Hou, Zhiyu Xun, Yang Liu, Jiyou Gu

**Affiliations:** 1 Key Laboratory of Bio-based Material Science and Technology of Ministry of Education, Northeast Forestry University, Harbin 150040, China; huopengfei@nefu.edu.cn (P.H.); 18846459163@163.com (S.N.); houpu13244521102@163.com (P.H.); 15963729607@163.com (Z.X.); liuyang@nefu.edu.cn (Y.L.); 2 Material Science and Engineering College, Northeast Forestry University, Harbin 150040, China

**Keywords:** soybean protein isolate, gel polymer electrolyte, crosslinked membranes, solid-state supercapacitor

## Abstract

A crosslinked membrane based on renewable, degradable and environmentally friendly soybean protein isolate was formed by solution casting method. A series of gel polymer electrolytes were prepared with the crosslinked membranes saturated with 1 mol·L^−1^ Li_2_SO_4_. The solid-state electric double-layer capacitors were fabricated with the prepared gel polymer electrolytes and activated carbon electrodes. The optimized solid-state supercapacitor delivered a single electrode specific capacitance of 115.17 F·g^−1^ at a current density of 1.0 A·g^−1^, which was higher than the supercapacitor assembled with the commercial separator in 1 mol·L^−1^ Li_2_SO_4_. The solid-state supercapacitor exhibited an outstanding cycling stability, indicating that the gel polymer electrolyte based on the crosslinked soybean protein isolate membrane could be a promising separator for a solid-state supercapacitor.

## 1. Introduction

Electric double layer capacitors (EDLCs) have emerged as high-performance energy storage devices for low cost, long operating lifetime and high power densities [1,2,3,4]. They have recently received increasing attention, and could significantly satisfy growing energy demands in our daily life. The performance of EDLCs strongly depends on electrode, electrolyte and separator. More attention has been payed to electrode materials and structures to improve electrochemical performance [5,6,7,8,9], although electrolytes and separators are still key components that can determine the maximum operating voltage, rate capability, lifetime and safety of EDLCs [10,11,12,13,14].

It is well known that most commercial EDLCs employ organic liquid electrolyte rather than aqueous electrolyte due to the much wider stable electrochemical potential window of the organic liquid electrolyte compared to the aqueous electrolyte (normally below 1.2 V). However, organic liquid electrolyte has several disadvantages due to its strict package and high flammability [15]. In comparison, aqueous electrolytes, in particular neutral aqueous electrolytes, have numerous advantages, such as a non-flammable nature, better corrosion resistance, greater safety, and they are also cheaper and more conductive [16,17,18]. However, to construct the solid-state supercapacitors used in miniaturized or flexible electronic devices, polymer electrolyte could be a unique replacement for conventional liquid electrolyte. Gel polymer electrolyte (GPE), one of the polymer electrolytes, can be prepared by mixing salt and solvent with the polymer, and deliver an acceptable ionic conductivity (10^−3^–10^−4^ S·cm^−1^). The application of GPE in the solid-state supercapacitor can solve the leakage problem as well as keeping the similar electrochemical performance of the supercapacitor assembled with a liquid electrolyte [19,20,21]. The choice and preparation of GPE is one of the key questions on designing a high-performance EDLC. Several polymers have been investigated as the matrix of the GPEs for EDLCs, such as poly (vinyl alcohol) (PVA) [22], poly (polyacrylate) (PAA) [23], poly (ethylene oxide) (PEO) [24], poly (vinylidene fluoride-co-hexafluoropropylene) (PVDF-HFP) [25], and poly (arylene ether) (PAE) [15], as well as their blends. However, most of the polymer mentioned above are synthetic polymers with hydrophobic main chains, which are generally neither renewable nor biodegradable, or need complex chemical modification to achieve better hydrophilicity.

Soybean protein isolate (SPI) is one of the biomass materials made out of a series of amino acids covalently linked through peptide bonds, which has been applied in food packaging material, medical material, biomass adhesives, composite material and other fields due to its many qualities. It is renewable, biodegradable, environmentally friendly, and has hydrophilic properties and reactivity. SPI has been investigated as one of the matrices for the membranes with ionic conductivity. For instance, a high performance soy protein-based solid conductor with a small amount of poly (ethylene oxide) (PEO) was prepared and showed an ionic conductivity of ~10^−5^ S·cm^−1^ [26]. Zhu prepared a series of porous membranes with SPI and poly(vinyl alcohol) (PVA) by using electrospinning technique [27]. The studies mentioned above demonstrate that SPI can be used for polymer electrolytes applied in electrochemical storage devices. However, there are few reports on the investigation of SPI applied in the field of solid-state supercapacitors.

In this work, the renewable and environmentally friendly SPI has been selected as the matrix material due to its inherent hydrophilicity without complicated modification. The application of SPI in supercapacitors further enhances the value of soybean protein, while also developing a novel kind of green material. However, the SPI based membranes applied in electrochemical storage devices mentioned above could not maintain a stable state in water for a long time without crosslinked structures, which would limit the application of SPI in supercapacitors due to the high water sensitivity, undesirable process ability, and the inferior mechanical strength of SPI [28]. In order to overcome the poor water resistance of SPI based membranes, a series of crosslinked membranes were prepared with SPI as the polymer matrix, crosslinked by ethylene glycol diglycidyl ether (EGDE), with a facile ring open reaction. EGDE is an epoxy compound with low toxicity [29,30]. The gel polymer electrolyte was designed by swelling the crosslinked SPI membrane into 1 mol·L^−1^ Li_2_SO_4_, which was then applied in a solid-state supercapacitor. The prepared gel polymer electrolyte could afford the solid-state supercapacitor a higher potential window than that of supercapacitors fabricated with acid or alkaline aqueous solution, for a higher energy density.

## 2. Experimental

### 2.1. Materials

SPI (96.5 wt %) was purchased from Harbin Gaoke Food Technology Co., Ltd. (Harbin, China). HEC was obtained from Aladdin Industrial Corporation (Shanghai, China). Glycerin, analytically pure, was acquired from Guangfu technology development Co., Ltd. (Tianjin, China). Activated carbon (AC) was provided by Japan KURARAY (Shanghai, China). Ketjen black (battery grade) was supplied by Crisco Chemical Technology Co., Ltd. (Shanghai, China). EGDE and Polytetrafluoroethylene (PTFE) (60 wt %) was purchased from Aladdin Reagent (Shanghai, China), Co., Ltd. Nickel foam was purchased from Kejing Zhida Co., Ltd. (Shenzhen, China). Anhydrous lithium sulfate and commercial hydrophilic PP/PE composite membrane were received from Saen Chemical Technology Co., Ltd. (Shanghai, China) and Lizhiyuan Battery Co., Ltd. (Shenzhen, China) respectively.

### 2.2. Preparation of SPI Membranes and GPEs

A casting solution was prepared by mixing the ethylene glycol diglycidyl ether (EGDE) as a crosslinking agent in a 5 wt % SPI/DI water solution with a small amount of glycerol/NaOH. Different mass fractions of the EGDE (0%, 20%, 40%, 60%, 80%) were introduced in the SPI/DI water solution, and further stirring for uniform mixing of the casting solution was carried out. The casting solution was coated on clean glass plates and completely dried at 50 °C in a vacuum oven for 24 h to remove DI water. The dried membranes were recorded as SPI/EGDE-*x* (*x* = 0, 0.2, 0.4, 0.6 and 0.8, which represented the EGDE for the mass fraction of the SPI).

**Figure 1:**
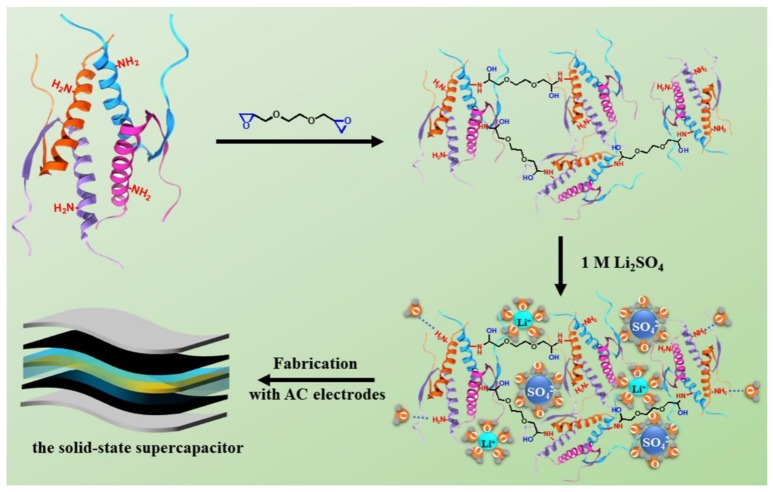
Schematic diagram of the construction process of the gel polymer electrolyte and the solid-state supercapacitor.

The GPEs were prepared by immersing the crosslinked SPI membranes into the 1 mol·L^−1^ Li_2_SO_4_ aqueous electrolyte for 2 h at room temperature, to trap the liquid electrolyte in the crosslinked SPI network for the formation of GPEs (Figure 1). The GPEs were named as GPE-*x* (*x* = 0%, 20%, 40%, 60% and 80%, which represented the EGDE for the mass fraction of the SPI).

### 2.3. Fabrication of the EDLC Single Cells with the GPE-x

The EDLC electrode was prepared using AC film and foamed nickel. Briefly, the AC films were prepared by mixing 80 wt % activated carbon, 10 wt % ketjen black and 10 wt % PTFE in ethanol, before being dried in a vacuum oven at 80 °C for 12 h. The EDLC single cell consisted of two symmetric AC electrodes sandwiching a piece of GPE-*x* film as shown in Figure 1. The assembled solid-state EDLC was sealed in an encapsulation cell and labeled as s-EDLC-*x* (*x* = 0, 20%, 40%, 60%, 80%).

### 2.4. Characterization

#### 2.4.1. FTIR

The ATR-FTIR spectra of the SPI/EGDE-*x* membranes were recorded on a Model Nicolette 6700 spectrometer (Thermo Scientific, Waltham, MA, USA) between 400 cm^−1^ and 4000 cm^−1^ using an ATR accessory.

#### 2.4.2. XRD

The X-ray diffraction (XRD) data of SPI and SPI/EGDE-20% were characterized by using X-ray diffraction equipment (Rigaku D/max220, Tokyo, Japan). The generator was set up at 40 kV and 30 mA, using Cu-Kα radiation (λ = 0.1542 nm) as the X-ray source, together with a Ni-filter to extract the Kα radiation. The data were collected over a range of scattering angles (2θ) of 5°–55°. All the measurements were carried out at room temperature under atmospheric pressure.

#### 2.4.3. Ionic Conductivity

The ionic conductivity of GPE-*x* was characterized by using a cell with a pair of 1.0 cm × 1.0 cm Nickel foam electrodes. The bulk resistance of GPE-*x* was measured by EIS measurement with ac potential amplitude of 10 mV and a frequency range from 1 Hz to 100 kHz at room temperature by using an electrochemical workstation (Model: CHI660A, Shanghai Chen Hua Co., Ltd., Shanghai, China). The ionic conductivity (*σ*, S·cm^−1^) could be calculated by the following equation:

(1) $$\sigma =\frac{L}{R_{b}\times S}$$

where *L* (cm) is the distance of the two electrodes, *S* (cm^2^) is the effective area, *R_b_* (ohm) is the bulk resistance of GPE-*x*, which is obtained from the EIS date.

#### 2.4.4. Electrochemical Performances of EDLC Single Cells

The electrochemical performances of the EDLCs were measured with electrochemical workstations (Model: CHI660A, Shanghai Chen Hua Co., Ltd. and LAND, Wuhan, China). The EIS measurements were performed in the frequency range of 0.01 Hz to 100 kHz and potential amplitude of 10 mV. The CV curves were obtained at various scan rates (5–200 mV·s^−1^) in a stable potential window of 0–1.5 V. The GCD measurements were carried out at various current densities (1.0, 2.0, 5.0 and 10.0 A·g^−1^) in a potential range of 0–1.5 V, the specific capacitances (*C_s_*, F·g^−1^) single electrode, energy density (*E_cell_*, W·h·kg^−1^) and power density (*P_cell_*, W·kg^−1^) of the EDLC single cell could be calculated depending on the GCD data by using the following equations:

(2) $$C_{s}=4\times \frac{I}{m\left(dV/dt\right)}$$

(3) $$E_{cell}=\frac{1}{2}C\Delta V^{2}=\left(\frac{C_{s}\times \Delta V^{2}}{8}\right)\times \frac{1000}{3600}$$

(4) $$P_{cell}=\frac{E_{cell}}{\Delta t}$$

where *m* (g) is the total weight of two electrode activated carbons, *I* (A) is the discharge current, and Δ*V* (V) is the potential change of the discharge process excluding the IR drop during the initial discharge stage. Δ*t* (h) is the discharge time.

## 3. Results and Discussion

### 3.1. ATR-FTIR Spectra of SPI Membranes

**Figure 2:**
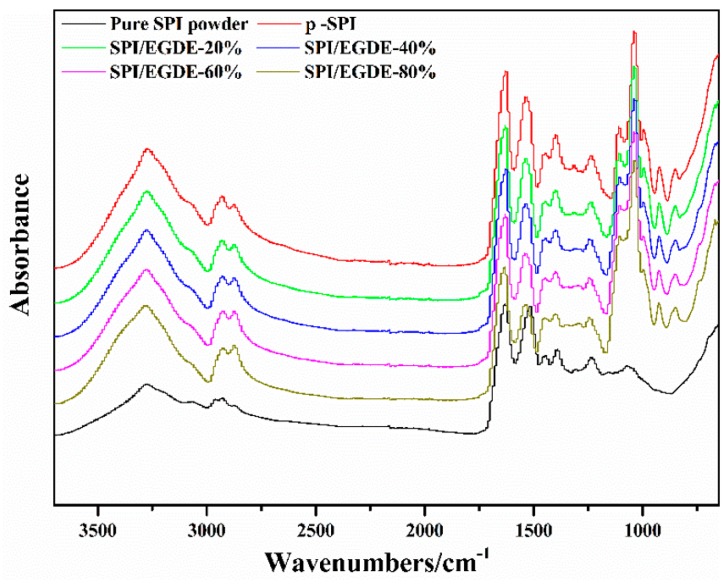
ATR-FTIR spectra of pure SPI powder and SPI membranes.

In order to understand the chemical structure of the SPI membranes, the pure SPI membrane and the crosslinked SPI/EGDE-*x* membranes were all analyzed by ATR-FTIR. The ATR-FTIR spectra of the SPI membranes showed the 1622 cm^−1^, 1530 cm^−1^ and 1232 cm^−1^ in Figure 2, which corresponded to amide I (C=O stretching), amide II (N–H bending) and amide III (C–N and N–H stretching), respectively [31]. The characteristic absorption peak of the epoxy group was not found in the infrared spectra of SPI/EGDE-*x*, indicating that the crosslinking reaction between EGDE and SPI had been carried out.

### 3.2. XRD of SPI Membranes

**Figure 3:**
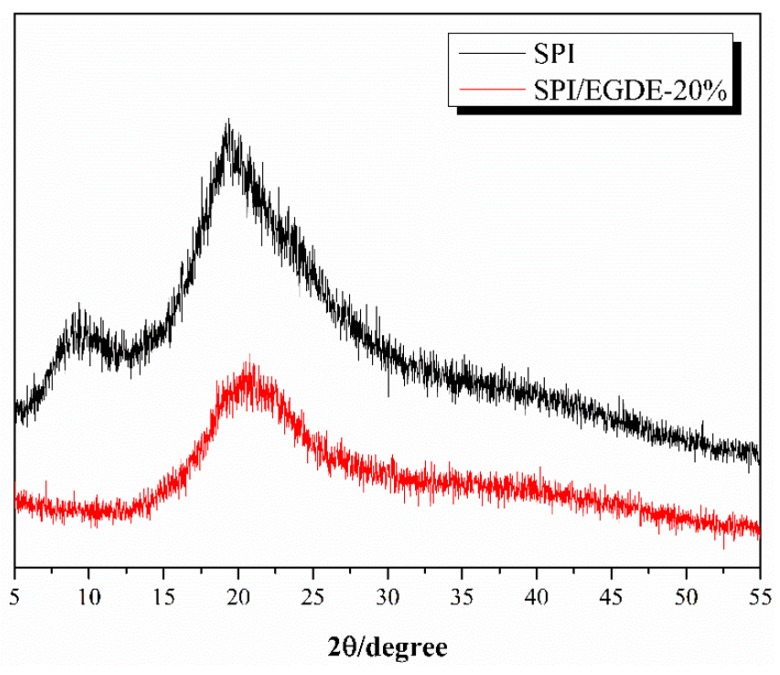
XRD patterns of SPI powder and SPI/EGDE-20% membrane.

Figure 3 shows the XRD patterns of SPI powder and SPI/EGDE membrane. The characteristic diffraction peaks at 2θ ≈ 9.1° and 19.2° of the pure SPI powder represent the α-helix and β-sheet secondary structures of SPI, respectively [32]. With the addition of EGDE, the characteristic absorption peak at 2θ ≈ 9.1° of SPI/EGDE membrane has disappeared, indicating that the chemical reaction has occurred between SPI and EGDE.

### 3.3. Ionic Conductivities of GPE

**Figure 4:**
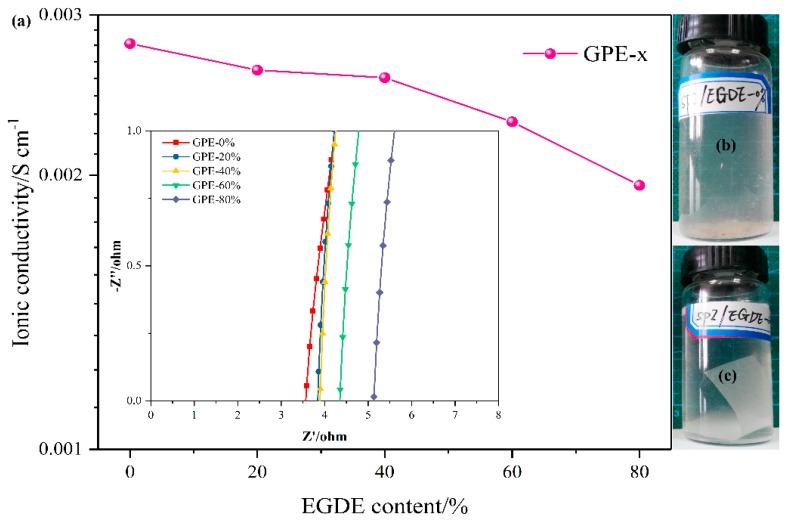
Ionic conductivities of GPE-*x* prepared by SPI/EGDE-*x* membranes (**a**) and water resistance of SPI/EGDE-*x* membranes (**b**,**c**).

The crosslinked structure dependence of the ionic conductivity of the GPE at room temperature is shown in Figure 4a. The GPE-*x* delivered the ionic conductivities ranging from 2.79 × 10^−3^ S·cm^−1^ to 1.95 × 10^−3^ S·cm^−1^. The excellent ionic conductivity of the GPEs were due to the good electrolyte affinity of the SPI/EGDE-*x* membrane. The crosslinked structure decreased the electrolyte uptake, resulting in a slight decrease of the ionic conductivity. However, the crosslinked structure enhanced the water resistance of the SPI/EGDE-*x* membrane. The crosslinked SPI membrane (SPI/EGDE-20%) was capable of maintaining excellent stability in the deionized water for 2 weeks, but the SPI membrane (SPI/EGDE-0%) dissolved in the deionized water after 2 days, as displayed in Figure 4b,c, which indicates that crosslinked structure can provide superior safety performance for the GPE and supercapacitor.

### 3.4. Electrochemical Properties of the Solid-State Supercapacitors

**Figure 5:**
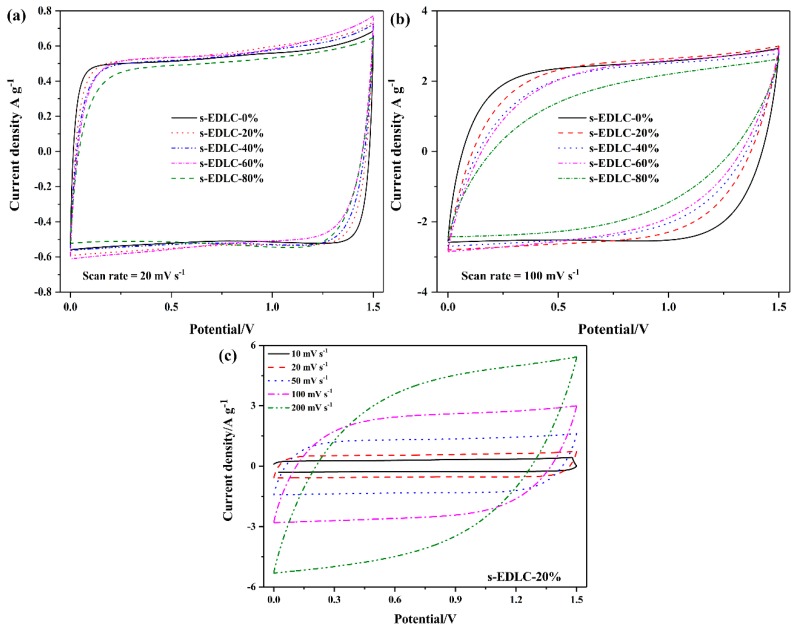
CV curves at a scan rate of 20 mV·s^−1^ (**a**) and 100 mV·s^−1^ (**b**) of the solid-state EDLCs and the CV curves of s-EDLC-20% at various scan rates (10–200 mV·s^−1^) (**c**).

**Figure 6:**
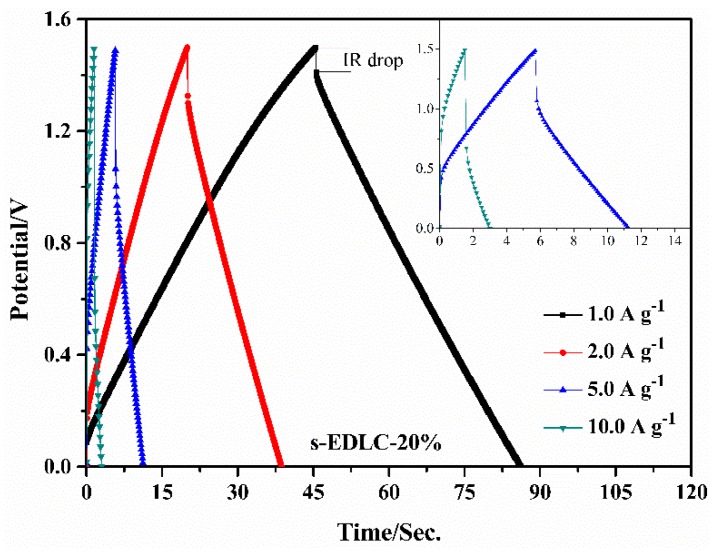
GCD profiles of the solid-state supercapacitor s-EDLC-20% at various current densities (1.0–10.0 A·g^−1^) with the GCD curves at 5.0 A·g^−1^ and 10.0 A·g^−1^ in the inset.

Figure 5a,b exhibits the CV curves of s-EDLC-*x* at 20 mV·s^−1^ and 100 mV·s^−1^, respectively. At the scan rate of 20 mV·s^−1^, all the solid-state supercapacitors show similar CV profiles close to the ideal rectangular shape without redox peaks, which reveals the ideal double-layer capacitance behavior of the AC based solid-state EDLCs [33]. However, at the high scan rate of 100 mV·s^−1^, the CV curves of the solid-state supercapacitors show that the higher the EGDE content is, the more the CV profile deviates from the ideal behavior. It is noted that the CV curve covering a larger current area at the same current density provides a higher capacitance for the supercapacitor [21,34]. The results prove that the lower cross-linking degree can result in improved electrochemical performance of the solid-state supercapacitor. The SPI/EGDE-0% membrane without crosslinked structure has poor water resistance, that the SPI/EGDE-0% membrane can be dissolved in deionized water within 48 h, but the SPI/EGDE-20% membrane with the crosslinked structure can maintain stability over a 2-week period, as shown in Figure 4b,c. The solid-state supercapacitor s-EDLC-20% shows the best CV behavior among the solid-state supercapacitors assembled with the crosslinked SPI/EGDE membranes. The CV profiles of s-EDLC-20% were investigated at various scan rates ranging from 10–200 mV·s^−1^ (Figure 5c). As the scan rate is elevated, the responding current increases, and the CV curve gradually deviates from the rectangular shape, but still maintains good electrochemical performance. As can be seen from the CV curves shown in Figure 5a–c, the solid-state supercapacitors can run stably within the higher working voltage of 1.5 V, when compared to those of the aqueous acid and base electrolyte (1.0 V). The GCD curves of s-EDLC-20% recorded from 0 V to 1.5 V at various current densities of 1.0–10.0 A·g^−1^ are illustrated in Figure 6. The GCD profiles are almost triangular at 1.0–5.0 A·g^−1^, which reveals the capacitive nature of the solid-state supercapacitors with the GPE-*x* up to 1.5 V, however, the GCD profile at a high current density of 10.0 A·g^−1^ slightly deviates from ideal behavior, with a larger IR drop due to the internal resistance resulting from the crosslinked structure.

**Figure 7:**
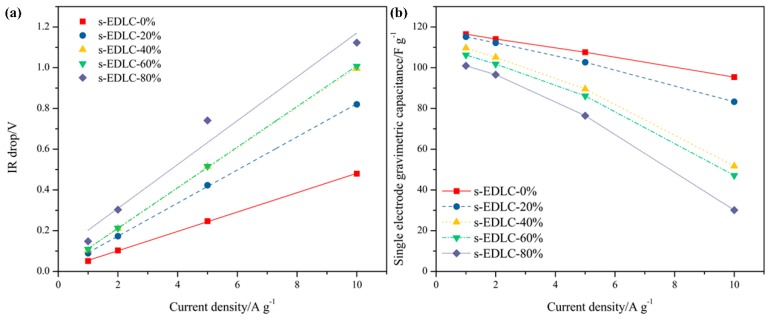
IR drops (**a**) and single electrode gravimetric capacitances (**b**) of the solid-state supercapacitors at various current densities.

The IR drops from GCD curves of all the solid-state supercapacitors at various current density are presented in Figure 7a. As can be observed, the IR drop increases with the current density. The solid state EDLCs exhibits a close IR drop at a small current density, such as 1.0 A·g^−1^, however, a larger IR drop of EDLC with more EGDE appears at a higher current density. This is due to the decreasing transportable ions in the polymer electrolyte resulting from the increasing content of EGDE. The single electrode gravimetric capacitances of all the solid-state EDLCs calculated from GCD curves are shown in Figure 7b. All the solid-state EDLCs exhibit high specific capacitances at a low current density of 1.0 A·g^−1^, however, as the current density increases, the solid-state EDLC assembled by the SPI membrane with higher EGDE content shows a more significant decrease in specific capacitance. The capacitance retention of s-EDLC-20% is 72.3% when the current density increases from 1.0 A·g^−1^ to 10.0 A·g^−1^, demonstrating a superior rate of performance benefiting from the high ionic conductivity of GPE-20%.

**Figure 8:**
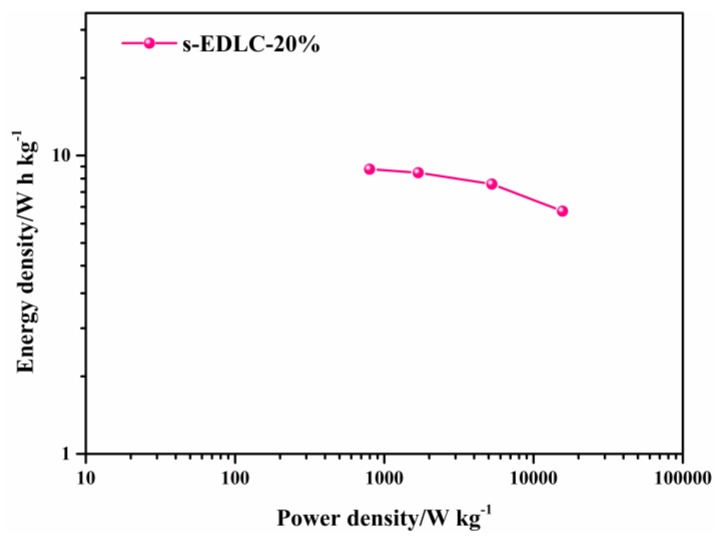
Ragone plot of solid-state supercapacitor s-EDLC-20%.

The Ragone plot of the solid-state supercapacitor s-EDLC-20% is exhibited in Figure 8. The highest energy density of s-EDLC-20% is 9.00 W·h·kg^−1^, delivered at a corresponding power density of 796.07 W·kg^−1^. The energy density still remains at 6.51 W·h·kg^−1^ at an ultrahigh power density of 15.62 kW·kg^−1^, indicating that the GPE-20% exhibits excellent electrochemical performance as a supercapacitor. The energy density is remarkably superior to that of previously reported carbon-based supercapacitors with polymer electrolyte based on aqueous electrolyte. Table 1 shows a comparison of the electrochemical performance of s-EDLC-20% with other supercapacitors assembled with AC electrodes. The supercapacitor s-ELDC-20% exhibits the highest energy density among the previously reported devices [15,35,36,37,38].

**Figure 9:**
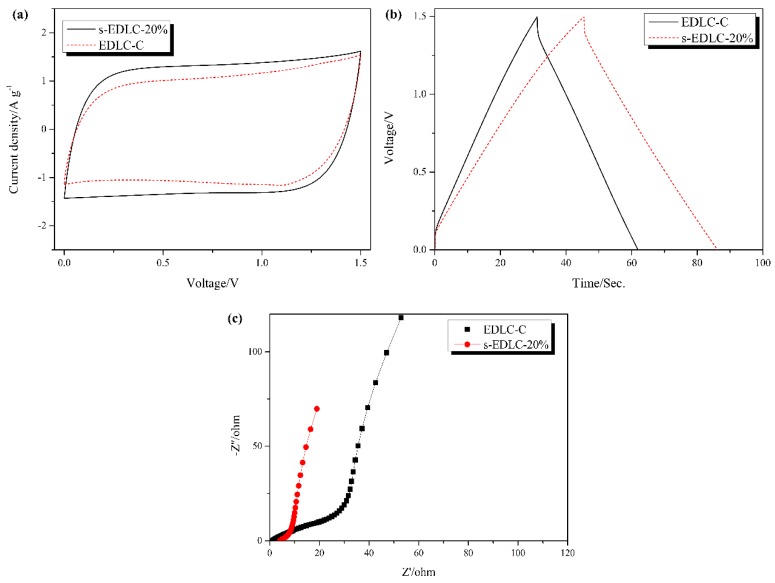
Comparison of CV curves (**a**), GCD profiles (**b**) and Nyquist plots (**c**) between s-EDLC-20% with the polymer electrolyte GPE-20% and EDLC-C with a commercial separator.

**Figure 10:**
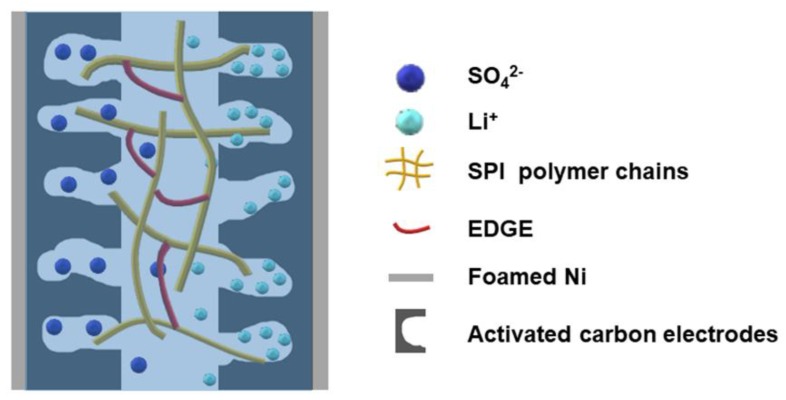
A simplified illustration of ion migration at the interface between the GPE and activated carbon electrodes.

The electrochemical performance of s-EDLC-20% with GPE-20% was compared to that of EDLC-C assembled with commercial hydrophilic PE/PP separator in 1 M Li_2_SO_4_ as depicted in Figure 9. The supercapacitor s-EDLC-20% shows a better CV profile at a scan rate of 50 mV·s^−1^. The GCD curves of s-EDLC-20% and EDLC-C show triangular profiles with a similar internal resistance drop, demonstrating a good electric double layer capacitance behavior. However, s-EDLC-20% delivers a single electrode specific capacitance of 115.17 F·g^−1^ higher than that of EDLC-C (86.22 F·g^−1^) at a current density of 1.0 A·g^−1^. The Nyquist plots of s-EDLC-20% and EDLC-C are shown in Figure 9c. At high frequency region, the first intersection between the Nyquist plot and Z’ axis denotes the equivalent series resistance (*R_b_*). An approximate semicircular behavior refers to the charge transfer resistance (*R_ct_*) related to the process at the GPE/electrode interface [37,39]. EDLC-C exhibit a lower *R_b_* than s-EDLC-20. However, Nyquist plots of s-EDLC-20% have a smaller depressed semicircle than that of EDLC-C. As the two supercapacitor devices use the same electrodes, the superior electrochemical performance of s-EDLC-20% could be because of the better interface compatibility. That is, the appropriate swelling behavior of GPE-20% greatly facilitates the Li^+^ ionic transport between the electrolyte and AC electrodes (Figure 10).

**Figure 11:**
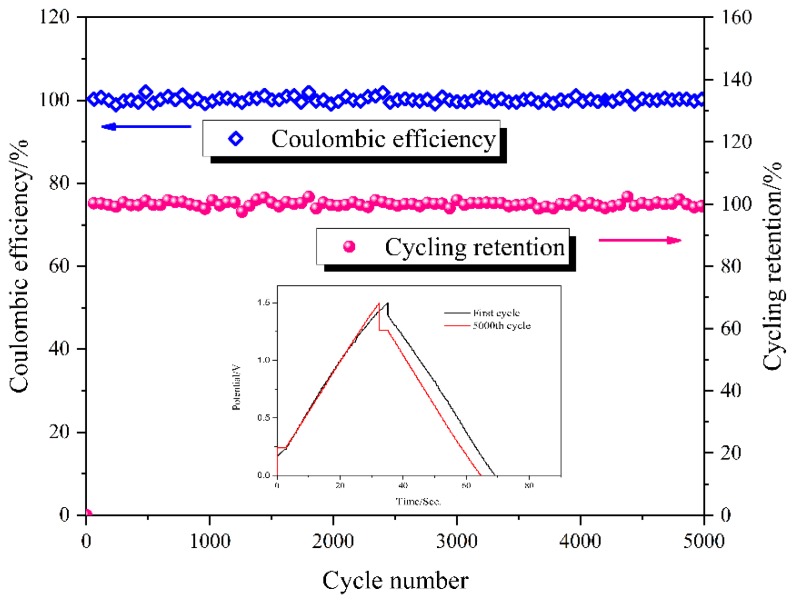
Coulombic efficiency and cycling retention of the solid-state supercapacitor s-EDLC-20% with the GCD profiles of first cycle and 5000th cycle in the inset.

The cycling stability of an electrochemical device is one of the key points for its practical application. To investigate the cycling stability, the solid-state supercapacitor s-EDLC-20% was tested by GCD technique at a constant current density of 1.0 A·g^−1^ over 5000 continuous cycles. The coulombic efficiency and cycling retention of the supercapacitor s-EDLC-20% for 5000 cycles are exhibited in Figure 11. The coulombic efficiency can be calculated from the ratio of the discharge capacitance to the charge capacitance, and remained above 95% for 5000 cycles, which indicates that s-EDLC-20% has good charge-discharge reversibility. The cycling retention remained close to 100% even after 5000 cycles as well. In the inset of Figure 11, The GCD profile of the 5000th cycle shows a slight deviation compared to that of the first cycle, which indicates that the solid-state supercapacitor s-EDLC-20% had good cycling stability. The results above demonstrate that the GPEs prepared by crosslinked SPI/EGDE membranes have excellent electrochemical and chemical stabilities.

## 4. Conclusions

In summary, a solid-state electrical double-layer capacitor was fabricated for the first time using a novel GPE-20% based on crosslinked SPI membrane and AC electrodes. The GPE-20% delivered a high ionic conductivity of 2.61 × 10^−3^ S·cm^−1^, an appropriate electrolyte uptake, good water resistance, and excellent electrochemical stability, suggesting suitability for solid-state supercapacitors. The fabricated s-EDLC-20% with GPE-20% exhibited good electrochemical performance. A high single electrode specific capacitance of 115.17 F·g^−1^ at a current density of 1.0 A·g^−1^ was obtained in a potential window of 0–1.5 V for an energy density of 9.00 W·h·kg^−1^. The resulting supercapacitor displayed a stable cycle performance.

We believe that supercapacitors and GPEs prepared with renewable and environmentally friendly biomass can increase the added value of biomass materials, reduce environmental pollution, simplify the preparation process of polymer electrolyte, and provide new ideas for the preparation of polymer electrolytes in electrochemical storage devices.

## Figures and Tables

**Table 1:** Performance comparison of s-EDLC-20% prepared in this work with the supercapacitors based on different polymer electrolytes from previous literatures.

Electrode Materials	Electrolytes	Specific Capacitance (*C_s_*)	Energy Density (*E_cell_*)	Operating Voltage	Reference
AC ^a^	SPI/EGDE/Li_2_SO_4_	115.17 F·g^−1^ at 1.0 A·g^−1^ (RT ^h^)	9.00 W·h·kg^−1^ at 1.0 A·g^−1^ (RT)	1.5	This work
AC	mCe-membrane/KOH ^c^	120.6 F·g^−1^ at 0.5 A·g^−1^ (RT)	4.37 W·h·kg^−1^ at 0.5 A·g^−1^ (RT)	1.0	[15]
AC	PAEK–40%PEG–LiClO_4_^d^	103.17 F·g^−1^ at 0.1 A·g^−1^ (120 °C)	6.76 at 0.1 A·g^−1^ (120 °C)	1.5	[35]
AC	PAES-Q/PVP/KOH ^e^	140.85 F·g^−1^ at 0.1 A·g^−1^ (RT)	4.81 W·h·kg^−1^ at 0.1 A·g^−1^ (RT)	1.0	[36]
RH-AC ^b^	EW-GPE-2 (NaCl) ^f^	214.3 F·g^−1^ at 0.2 A·g^−1^ (RT)	4.76 W·h·kg^−1^ at 0.2 A·g^−1^ (RT)	0.8	[37]
AC	PAES-Q-1.1 ^g^	92.79 F·g^−1^ at 0.1 A·g^−1^ (RT)	2.61 W·h·kg^−1^ at 0.1 A· g^−1^ (RT)	1.0	[38]

^a^ AC represents commercial activated carbon; ^b^ RH-AC represents activated carbon based on rice husk; ^c^ mCe-membrane/KOH is prepared by porous cellulose membrane with 6 mol·L^−1^ KOH; ^d^ PAEK-40%PEG-LiClO_4_ is prepared by poly(aryl ether ketone)-poly(ethylene glycol) copolymers with LiClO_4_; ^e^ PAES-Q/PVP/KOH is prepared by quaternary ammonium functionalized poly(arylene ether sulfone) copolymers and polyvinylpyrrolidone saturated with 6 mol·L^−1^ KOH; ^f^ EW-GPE-2 (NaCl) is prepared by absorbing the necessary amount of egg white electrolyte (with NaCl) on to the eggshell membrane; ^g^ PAES-Q-1.1 is prepared by quaternary ammonium functionalized poly(arylene ether sulfone) copolymers; ^h^ RT represents room temperature.
